# Reconstruction of mandibular defects using vascularized fibular osteomyocutaneous flap combined with nonvascularized fibular flap

**DOI:** 10.4317/medoral.23040

**Published:** 2019-08-19

**Authors:** Weihong Wang, Jin Zhu, Biao Xu, Bin Xia, Yu Liu, Shengjie Shao

**Affiliations:** 1 Department of Oral and Maxillofacial Surgery, Affiliated Stomatology Hospital of Kunming Medical University, Kunming 650106, China

## Abstract

**Background:**

The height of single-layer fibular flap is not long enough to return to the ideal height of the mandible. While the double-layer vascularized fibular osteomyocutaneous flap(VFF) is more complicated in shaping and fixation, along with a longer operation time. The aim of this study was to investigate the clinical effect of VFF combined with nonvascularized fibular flap(NVFF) in the reconstruction of mandibular defect.

**Material and Methods:**

From September 2016 to June 2018, 15 patients with benign mandibular tumors underwent reconstruction with VFF and NVFF. SimPlant Pro ™ software (version 11.04) was used to simulate reconstruction of the mandible preoperatively.

**Results:**

All patients were followed up for 8-23 month, with an average of 11.7 months. 15 VFFs survived well. Among the 15 NVFFs, one was almost completely absorbed, two with partial absorption, and the remaining survived regardless of the small amount of absorption. The postoperative absorption of the whole fibula was 7.53±6.362%, a favorable facial contour and speech function were attained.

**Conclusions:**

The VFF combined with NVFF to reconstruct the mandibular defect can restore the vertical height of the mandible and achieve satisfactory clinical results.

** Key words:**Vascularized fibular osteomyocutaneous flap(VFF), Nonvascularized fibular flap(NVFF), Mandibular defect.

## Introduction

Large segmental mandibular defects frequently lead to severe facial deformities and result in difficulty in chewing, which have seriously affected the quality of life of patients. Therefore, it is now advocated that the vascularized fibular osteomyocutaneous flap(VFF) should be used to reconstruct the mandible and restore the facial and chewing functions of the patients with the large segmental mandibular defect (1-3). However, the height of single-layer fibular flap is not long enough to return to the ideal height of the mandible. Thus, the vertical difference between the single-layer fibular flap and the maxillary occlusal plane would adversely affect the stability and retention of the later denture. By contrast, the distraction osteogenesis (DO) technology can improve the height of the mandible. Nonetheless, the long treatment during of DO bring about more complications, such as local chronic infection and distraction of the distractor (4). On the other hand, fixing the fibular flap in the middle or the upper edge of the mandibular stump is beneficial for later denture repair (5). However, such method would not fully restore the facial symmetry. Notably, reconstructing the mandibular defect with a double-barrel vascularized fibular osteomyocutaneous flap can completely restore the vertical height of the mandible. Moreover, such method contributes to the favorable facial contour, few complications, and satisfactory clinical efficacy in patients (6-8). Nevertheless, a longer fibula is required for the double-barrel vascularized fibular osteomyocutaneous flap, which is not conducive to the postoperative stability of the lower limb. Besides, the double-layer vascularized fibular osteomyocutaneous flap is more complicated in shaping and fixation, along with a longer operation time. Furthermore, the vascular pedicle becomes shorter after the fibular flap of the transplant is folded, which will add to the difficulty in anastomosis, especially for the large-section LC type mandibular defect (6-8). To overcome the above shortcomings, 15 patients with benign mandibular tumors were reconstructed with the vascularized fibular flap combined with the nonvascularized fibular flap(NVFF) following tumor resection. The preoperative virtual surgery was performed using the SimPlant ProTM software, and satisfactory clinical results were achieved.

## Material and Methods

From September 2016 to June 2018, 15 patients with benign mandibular tumors-including 10 males and 5 females, were admitted to the Department of Oral and Maxillofacial Surgery, Affiliated Stomatological Hospital of Kunming Medical University. The age of patients ranged from 20 to 64 years, with an average of 41.7 years. 11 of the 15 patients presented with ameloblastomas, 2 presented with myxomas, 1 presented with ossifying fibroma, and 1 presented with inflammatory myofibroblastic tumor. According to the classification of mandible (8), 2 were type C defects (the entire central segment, including both lower canines), 7 were type H defects (the lateral segment including the condyle), 2 were type L defects (a lateral segment that does not include the condyle) and 4 were type HC defects (including type H and C). This study was approved by the Human Science Committee of Kunming Medical University. The VFF and the NVFF were used to reconstruct the defect following mandibular resection. The YZB2.0 craniofacial fixation system (Shenzhen Putianyang Medical Instrument Co., Ltd) was used to reconstruct the titanium plate and screws for fixation. Clinical data of the 15 patients are presented in  Table 1.

**Table 1:**
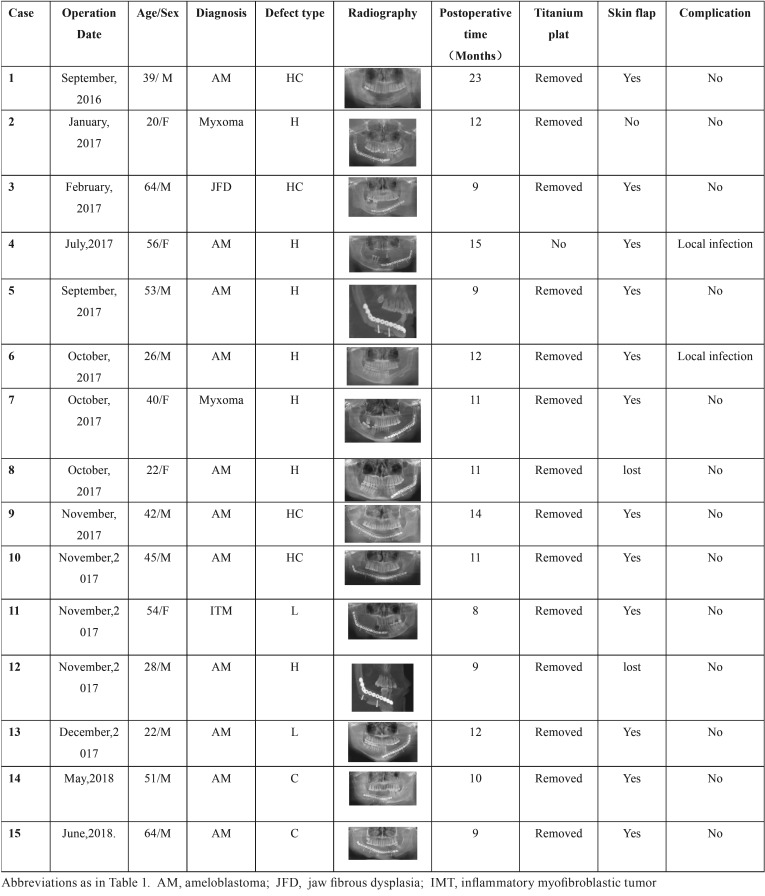
Clinical features of 15 patients with benign mandibular tumors.

**Figure 1:**
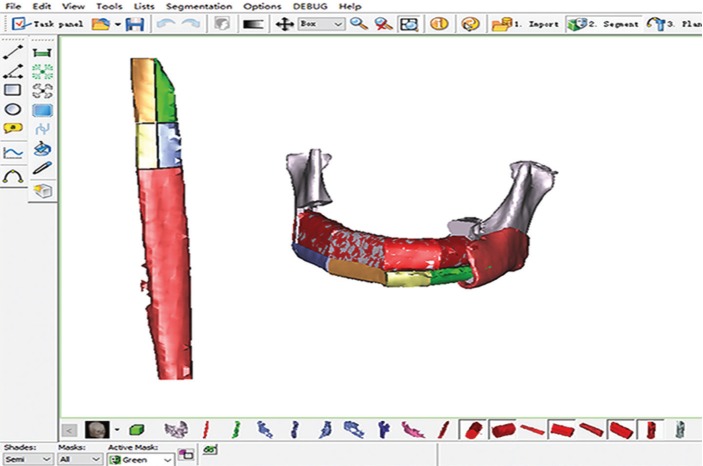
Three-dimensional virtual surgery for the reconstruction of mandibular defects using vascularized fibular flap combined with nonvascularized fibular flap.

**Figure 2:**
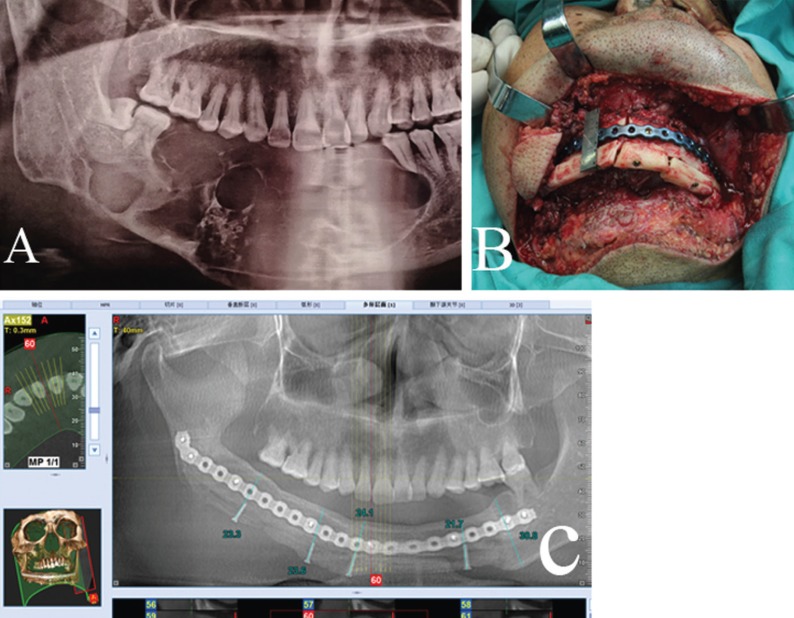
A. Preoperative panoramic radiograph showed a mandibular ameloblastoma. B. The nonvascularized fibular flap has been sagittalized and segmented, make a small groove at the lower edge, and then fix it to the upper vascularized fibular flap with a 2.0×10.0mm titanium screw. C. Postoperative CBCT showed the vascularized and nonvascularized fibular flap survived well 14 months later.

CT scans were performed preoperatively, and the SimPlant ProTM software (version 11.04, Materialise NV, Leuven, Belgium) was used to simulate the mandibular reconstruction, as described in our previous study (Fig. 1) (7). The mandible was exposed under general anaesthesia using nasoendotracheal intubation, the tumor was then completely removed, and the oral mucosa and submucosal tissue were subsequently sutured. Later, the muscles surrounding the fibula were dissected with using an ultrasonic-harmonic scalpel after the fibula was exposes without the use of a tourniquet. VFF was harvested near the lower part of the fibula, whereas the NVFF was obtained near the upper end of the fibula, as well as the perforators. Typically, the adhesion of the flexor hallucis longus to the upper fibula was reserved. Afterwards, the harvested fibula was shipped and fixed to the reconstructed titanium plate, and, the pedicle was not dissected from the donor site until the recipient vessels were prepared. Then, both the vascularized fibular flap and the reconstructed titanium plate were transferred to the recipient site and fixed onto the upper edge of the mandibular stump, and the blood vessels were anastomosed. Subsequently, the harvested NVFF was sagittalized from the middle and segmented based on the shape of the VFF as well as its relationship with the mandibular stump. At the same time, the NVFFs that had been sagittalized and segmented were trimmed. Afterwards, a small groove was made at the lower edge, and fix onto the upper VFF with a 2.0×10.0mm titanium screw (Fig. 2). Finally, two suction tubes were conventionally placed. Besides, all patients had undergone a tomographic or CBCT examination during the follow-up period.

The age and gender of patients, tumor location and pathological diagnosis were analyzed using the IBM SPSS software package (version 21, IBM Corp, Armonk, NY). The vertical height of the neo-mandible from the intraoperative measure and postoperative CBCT at the last follow-up visit (Fig. 2) was compared using the paired t-test, and the mean value was expressed in SD. Probabilities of less than 0.05 were deemed as significant.

## Results

**Figure 3:**
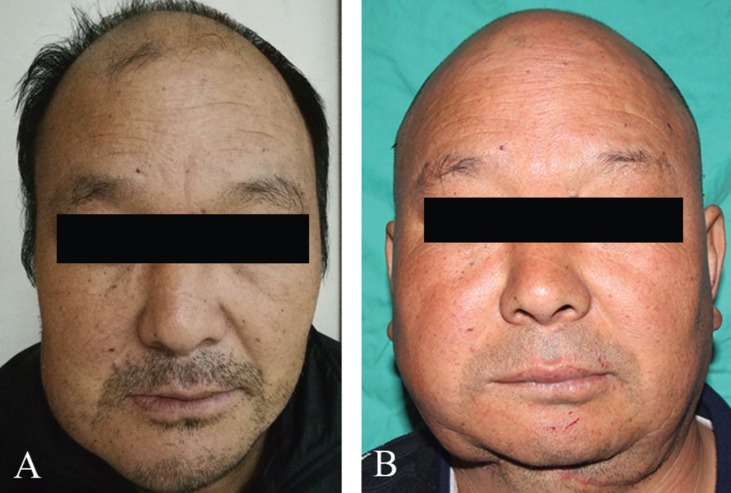
A. The preoperative front view; B. The postoperative front view 14 month later.

All patients had no blood transfusion intraoperatively except one who developed a venous crisis of the perforator flap on the second day after surgery, and 400 mL whole blood was delivered during the second operation. Additionally, two out of the 15 patients suffered from chronic infection at the submandibular region after the removal of the vacuum drainage device on the fifth days after surgery, which healed after one week of continuous dressing change, while the remaining 13 patients healed well at the incision site. Of the 12 perforator flaps, 10 survived completely; however, the remaining two, which were used to reconstruct the L-shaped mandibular defects, encountered with the venous crisis of the flap on the second day after surgery. Notably, intraoperative exploration revealed that the perforating vascular pedicle was locally compressed; as a result, so the flap was removed. All patients were followed up for 8-23 months, with an average of 11.7 months. Postoperative examination indicated that 15 VFFs survived well. In contrast, among the 15 NVFFs, one was almost completely absorbed, two with partial absorption, and the remaining survived regardless of the small amount of absorption. The original, intraoperative and postoperative vertical heights of the mandible were 25.62±4.00mm, 20.17±2.76mm and 18.68±3.06mm, respectively, the postoperative absorption of the whole fibula was 7.53±6.36%, all reconstructed mandible had a high vertical height and a favorable facial contour (Figs. 2,3), except for one patient who suffered from severe absorption of the NVFF. So far, 14 cases of the reconstructed titanium plate and screws were removed, and there was no conspicuous difference between the neomandibles reconstructed with VFF and that with NVFF. Notably, there was no obvious dysfunction in the ankle joint, even though there were obvious scars in the calf area of all patients.

## Discussion

Most of the bone defects reconstructed using NVFF transplantation are limb bones, such as poor healing of scaphoid fracture (9), ankle joint (10), discontinuous femoral neck fracture (11), and defect of giant cell tumor in distal radius (12). Jeong et al (10) successfully repaired one ankle joint with a 15 cm NVFF. Giordano et al (13) harvested a 21 cm of NVFF and divided it into 9 cm and 12 cm approximately, which were then used to successfully repair the distal femoral condyle defect in a 86-year-old patient caused by a comminuted fracture of the distal femur. Compared with vascularized fibular graft, NVFF is associated with a certain risk of failure, but the above-mentioned successful cases also indicate that, it is feasible to the reconstruct bone defects using NVFF in the clinic (14).

Nonvascularized rib and fibular graft are the common donors for reconstructing the mandibular defect, in the presence of no or little soft tissue defect (3). Lee *et al.* (15) fixed the VFF to the mandibular base, sagittalized the residual nonvascularized fibula, and placed it onto the VFF to reconstruct the alveolar bone, which was proved to improve the height of the reconstructed mandible and correct the vertical difference. However, the NVFF was used as the upper bone mass to increase the alveolar ridge height, which was linked with a relatively weak anti-infective ability in this surgical procedure. Typically, a decohesion in the oral mucosa would likely cause failure of the above nonvascularized fibula transplantation (14). In the current study, primary tumor resection and fibular flap preparation were performed simultaneously, and oral mucosa and submucosal tissue were sutured immediately after tumor resection by a team. At the same time, the harvested fibula was completed by another team who transferred the vascularized fibular flap to the recipient site and fixed it to the mandible. Subsequently, the shaped nonvascularized fibula segment was fixed below the vascularized fibula. Such surgical procedure can save the operation time compared with the double-barrel vascularized fibula flap descripted in our previous report(7). Moreover, the aesthetic and late denture repair would not be seriously affected even in the presence of the absorption or failure of the underlying NVFF, so long as the upper VFF survives. In this study, only two patients developed local infection, and the two NVFF segments were partially absorbed, but the patients appearance were not seriously affected. Unfortunately, the NVFF segment in one patient with LC-type mandibular defect was almost completely absorbed after surgery(case 10 in  Table 1), which may be due to the longer time that the nonvascularized fibula segment leaves the donor site. In our previous patient series, the NVFF segment was fixed under the vascularized one before the fibular vessel was broken, and the whole fibula segment was then transferred to the recipient site. But our results suggested that thus method was not convenient for fixing the entire fibula flap to the mandible, since the NVFF below might not be suitable when the upper VFF was fixed in the position, which would result in the prolonged operation time. Therefore, it is recommended that the VFF should be fixed to the mandible first prior to harvesting and immediately shaping the NVFF segment, which was then fixed below the VFF. This could not only save the operation time, but could also reduce the incidence of nonvascularized bone segment away from the donor site, which might therefore have a certain preventive effect on reducing its absorption. In this study, patients were followed up for 8-23 months, with an average of 11.7 months. The average height of the reconstructed mandible (18.68±3.06 mm) is sufficient for future implant denture repair, although its height is lower than that of the original mandibular body (25.62±4.00 mm). The postoperative absorption of the whole fibula was 7.53±6.36%, which was consistent with that reported in literature (13.6±7.2% of the nonvascularized fraction and 3.0±3.7% of vascularized fraction) (15). Accordingly, satisfactory postoperative facial contour was attained.

In this study, the NVFF was harvested from the upper end of the fibula as much as possible, which was half of that of the mandibular body defect in the length, so that the periosteum at the upper end of the fibula could be preserved, This could thereby preserve the adhesion of the flexor hallucis longus at the upper end of the fibula, prevent the flexor hallucis longus from the postoperative dysfunction expected in this study, and also lengthen the vascular pedicle length of the vascularized fibular flap. In addition, no blood transfusion was performed among all patients during fibular graft preparation even without the use of a tourniquet, which could indeed contribute to the reduced bleeding and the clear anatomical field of view. However, more local bleeding was observed after the tourniquet was released, which could hardly be stopped and might increase the risk of postoperative complications in the donor site (16,17). On this account, it is recommended to use an ultrasonic-harmonic scalpel to dissect the muscles around the fibula, which can reduce bleeding and save the operation time (18). In addition, for patients with L-shaped mandibular defects, the use of lower end of the fibula to reconstruct the mandibular ramus and the placement of the perforator flap behind such mandibular ramus may increase the risk of the venous crisis of the flap. Among all patients in this study, two were unfortunately encountered with the venous crisis of the flap two days after surgery, which was resulted from the above situation. Thus, the use of the flap should be decided according to the specific position of the perforator vascular pedicle for patients with L-shaped mandibular defects.

In summary, employing VFF combined with NVFF to reconstruct the mandibular defect can restore the vertical height of the mandible and achieve satisfactory clinical results. However, follow-up study should be necessarily carried out to examine whether there is continued absorption in the nonvascularized fibular part of the long term. Also, a single or double-barrel vascularized fibular flap is recommended to use for mandibular defects caused by malignant tumors (6,7).
